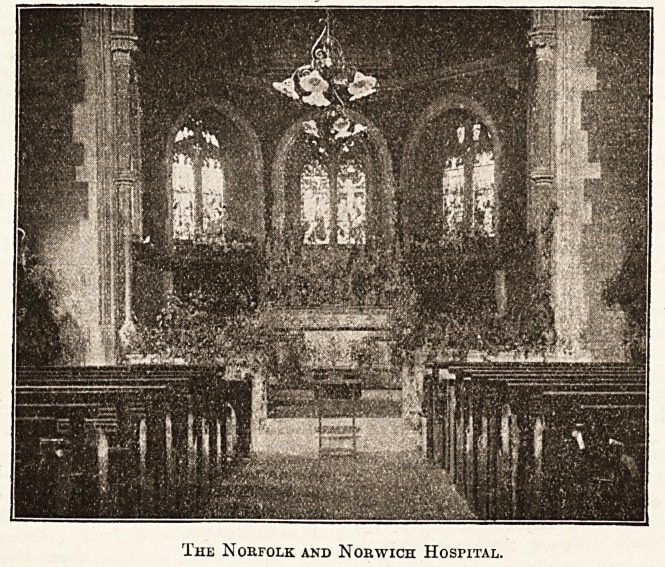# Some Provincial Hospital Chapels
*Previous Articles Appeared on January 23 and 30.


**Published:** 1915-02-13

**Authors:** 


### III.—SOME PROVINCIAL HOSPITAL CHAPELS.*

### Norfolk and Norwich Hospital.

The chapel of the Norfolk and Norwich Hospital, though possessing little or no history, being only built in 1883 as the result of public subscription, is none the less a building worthy in design and furniture for its sacred purpose; it is in the Gothic style, of red brick with stone facings, and consists of a nave lighted by six windows, with a semi-octagonal chancel, which possesses five windows, these in each case being of stained glass and given by various donors "in memoriam"; there is also an excellent little organ. The Rev. J. Huxley is the Chaplain to the institution.

The Norfolk and Norwich Hospital.

### A Byzantine Chapel at Newcastle.

The chapel of the Royal Victoria Infirmary, Newcastle, dedicated to St. Luke, and which was consecrated on September 19, 1906, by the Lord Bishop of Newcastle, the Right Rev. Arthur T. Lloyd, is in the form of a Greek cross with arched and domed roof, and is a unique example of Byzantine design, the faience-lined walls and dome and arches faced with mosaic being also in strict keeping with this Eastern character. The chapel has seating accommodation for 120 persons, and is fittingly furnished by possessing as memorials to departed friends or benefactors of the infirmary a beautiful carved reredos, pulpit, reading-desk, lectern, and organ, while an air of sanctity and rest is secured by the reflected light of its stained-glass windows. The Rev. F. I. Wilcox is the Chaplain to the institution.

### A Remarkable Triptych at Nottingham.

Built in the year 1853-1854, the chapel of the General Hospital, Nottingham, has accommodation for 150; it is built in the Gothic style, of brick with stone facings. Perhaps the most noteworthy feature is a triptych in oils, the gift of Sir Charles Seely, at the east end of the chapel, the panels representing—in the centre—the Visit of the Magi, while on either side respectively are St. Cecelia and St. Dorothea. A small but suitable organ is situated at the west end. The Rev. C. Davis is the Chaplain to the institution.

* Previous articles appeared on January 23 and 30.

## Figures and Tables

**Figure f1:**